# A patient with concurrent Axenfeld-Rieger and Stickler syndromes verified by molecular genetics

**DOI:** 10.1016/j.ajoc.2023.101931

**Published:** 2023-09-26

**Authors:** Jason Fan, Natasha Ferreira Santos da Cruz, Kenneth C. Fan, Catherin I. Negron, Guillermo Amescua, Alana L. Grajewski, Ta C. Chang, Audina M. Berrocal

**Affiliations:** Department of Ophthalmology, Bascom Palmer Eye Institute, Miami, FL, USA

**Keywords:** Axenfeld-Rieger Syndrome, Stickler syndrome, Retinal detachment, Anterior segment dysgenesis

## Abstract

**Purpose:**

To report a case of Axenfeld-Rieger and Stickler Syndrome in a pediatric patient.

**Observations:**

A 3-month-old male was referred to the glaucoma clinic after he was noted to have elevated intraocular pressures in both eyes. His family history was notable for infantile glaucoma on his maternal side and retinal detachment on his paternal side. He was found to have anterior segment dysgenesis with iris strands, iridocorneal adhesions, and corectopia, as well as veil-like vitreous in both eyes. He required trabeculotomy, goniotomy, and multiple Baerveldt glaucoma implants in both eyes to achieve intraocular pressure control. Furthermore, the patient later developed macula-involving retinal detachments in both eyes, requiring pars plana vitrectomy with silicone oil tamponade. Genetic analysis confirmed heterozygous pathogenic variants in both the *FOXC1* and *COL2A1* genes, leading to the concurrent diagnoses of Axenfeld-Rieger and Stickler syndromes.

**Conclusions and importance:**

This is a rare case of a patient with concurrent Axenfeld-Rieger and Stickler syndromes. The severity of pathology in both the anterior and posterior segments required a collaborative multidisciplinary approach. In the diagnostic evaluation of congenital eye diseases, if there is strong family history of atypical findings for a given diagnosis, concurrent syndromes should be considered and ruled out. A comprehensive eye genetics panel may be a useful tool in these cases.

## 1 Introduction

Axenfeld-Rieger Syndrome (ARS) is a rare multi-system congenital disorder that affects 1 in 200,000 live births.^1^ Systemically, ARS may cause cardiovascular outflow tract, craniofacial, skeletal, and neuroendocrine abnormalities. Ophthalmologic features of ARS include anterior displacement of Schwalbe's line (posterior embryotoxon), iris strands with iridocorneal adhesion (Axenfeld Anomaly), and iris hypoplasia resulting in corectopia. However, ARS exists within a spectrum of anterior segment dysgenesis, and there may be variable expressivity even within well-defined pedigrees.^2^ The first and most well-characterized causative mutations are within the *PITX2* and *FOXC1* genes, which account for 40–70% of cases.^3^^,^^4^

Glaucoma may develop in 50% of ARS cases and is thought to occur due to abnormal neural crest migration, leading to persistent endothelial cell coverage of the trabecular meshwork, anterior iris root insertion, and abnormal trabecular meshwork morphology.^2^ The development of glaucoma appears to be correlated more with the presence of an anterior iris root insertion rather than the iridocorneal adhesions.^5^ Rarely, patients with ARS may also have posterior pathology: retinal detachment was described in 3 members of a family with ARS.^6^

Stickler syndrome (hereditary arthro-ophthalmopathy) is an inherited connective tissue disorder, and the most common cause of retinal detachment in the pediatric population. The incidence is estimated to be 1 in 7500 to 9000 live births, and patients may also have systemic manifestations of connective tissue disease such as cleft palate, Pierre-Robin sequence, malar hypoplasia, hearing loss, and joint disease.^7^ Ophthalmologic manifestations may include abnormal vitreous, myopia, megalophthalmos, radial perivascular lattice degeneration, and retinal detachment. There are 5 subtypes, each with mutations in separate collagen genes. Type 1, the most common subtype, is associated with pathogenic *COL2A1* variants and has an autosomal dominant inheritance. Genes in collagen IX (*COL9A1*, *COL9A2*) and XI (*COL11A1*, *COL11A2*) constitute the other subtypes, and may have a recessive inheritance.^8^ Rarely, some patients may also have anterior segment dysgenesis leading to glaucoma.^9^, ^10^, ^11^

Although ARS and Stickler syndrome patients have overlapping clinical features, to our knowledge, there are no reported cases of concurrent Axenfeld-Rieger and Stickler syndromes confirmed by molecular genetics.

## 2 Case report

A 3-month-old Hispanic male was referred to the ophthalmology clinic after his pediatrician noted a poor red reflex in both eyes. He was born at a gestational age of 39 weeks with a birthweight of 3.58 kg. His birth was uncomplicated, and he received no supplemental oxygen or antibiotics. Family history was notable for infantile-onset glaucoma in his mother, maternal grandfather, and maternal great uncle, as well as high myopia and retinal detachment in his father. His mother and father had met at a school for the blind.

On examination, the patient's vision at near was central, steady, and maintained in each eye. He had intraocular pressures (IOP) of 50 mmHg in his right eye (OD) and 60 mmHg in his left (OS) by rebound tonometry (on no topical nor systemic IOP-lowering agents). Pupils were minimally reactive but there was no obvious relative afferent pupillary defect. He was not photophobic and did not have excessive tearing or blepharospasm. Anterior exam disclosed enlarged and clear corneas without obvious striae.

**Fig. 1:**
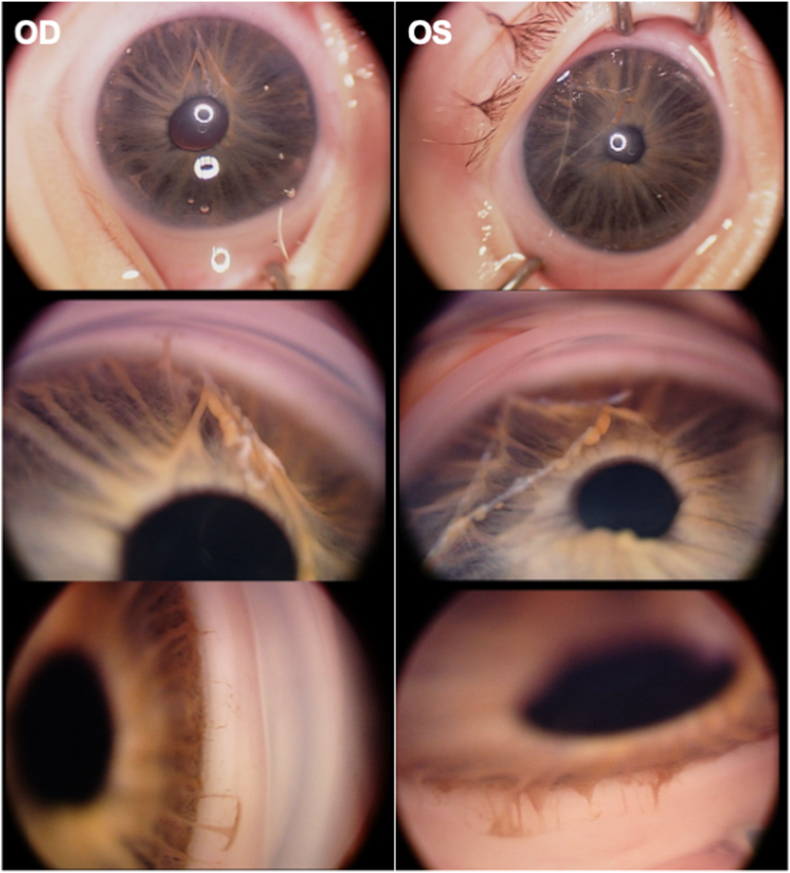
Intra-operative external and gonioscopy photos of the right and left eyes demonstrating iris strands, corectopia of the right eye, and abnormal iridocorneal adhesions in both eyes.

**Fig. 2:**
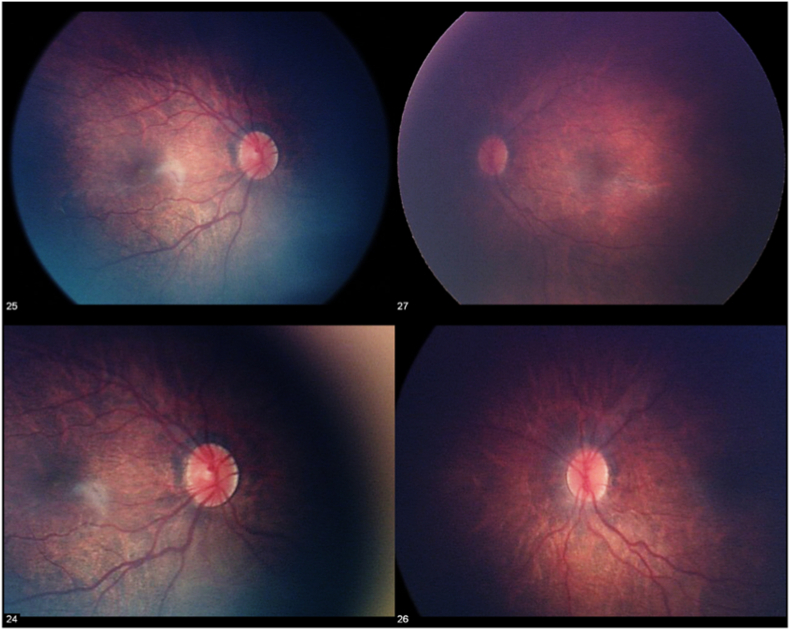
Fundus photography showing elongated discs, peripapillary atrophy, and pigmentary mottling in both eyes.

An exam under anesthesia was performed which showed iris strands with iridocorneal adhesions in both eyes (OU) and corectopia of the right eye (Fig. 1). Posterior exam was notable for elongated and tilted optic nerves with peripapillary atrophy, posterior staphylomas, and pigmentary mottling OU (Fig. 2). IOP was 30 mmHg OU on topical dorzolamide and betaxolol. Axial lengths measured by echography were 23.9mm OD and 24.1mm OS, both greater than 2 standard deviations above the mean (19.4mm) for this age. Bilateral Harms trabeculotomies were performed and the patient had an uncomplicated post-operative course. Over the next 6 months, the child had biometric changes consistent with progressive glaucomatous damage, and multiple additional angle surgeries and Baerveldt glaucoma drainage implants were required for IOP control.

At 1 year of age, the patient developed a visually significant cataract in the left eye and underwent uneventful pars plana lensectomy, vitrectomy, fluid-air exchange, and endolaser with planned aphakia. At 3 years of age, a macula-involving inferotemporal retinal detachment was noted OS on exam and B-scan ultrasonography. A combined vitrectomy with second Baerveldt implant was performed OS. A second Baerveldt implant was inserted because of elevated IOP and worsening optic nerve cupping despite topical dorzolamide and betaxolol therapy twice daily.

**Fig. 3:**
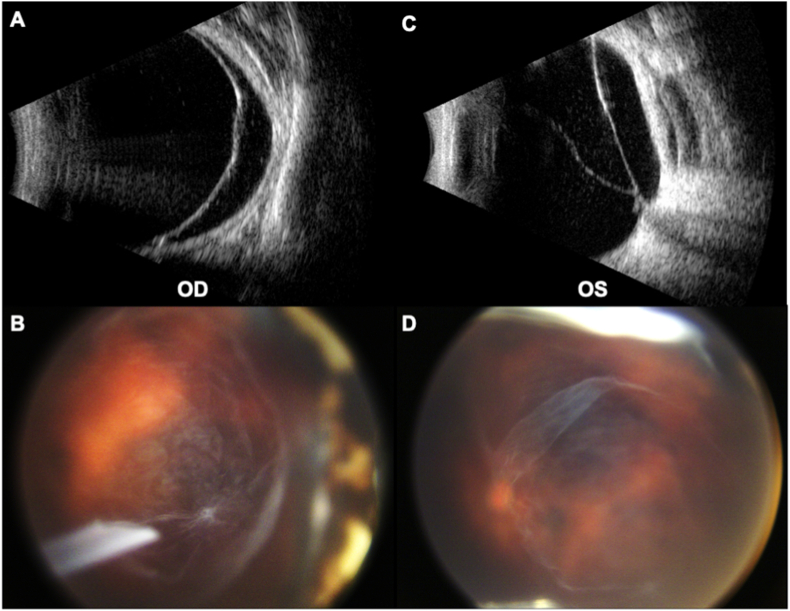
B-scan ultrasonography of demonstrating retinal detachment in the right eye (A) and left (C) eyes at the time of their diagnosis. Fundus photography demonstrating vitreous veils in the right (B) and left (D) eyes.

At 5 years of age, a new macula-involving retinal detachment was noted on B-scan ultrasonography OD (Fig. 3A “OD”). The patient underwent pars plana vitrectomy with membrane peel, endolaser, and silicone oil tamponade. Abnormal vitreoretinal interface and veil-like vitreous strands were noted intraoperatively (Fig. 3B, D). His post-surgical course was complicated by corneal failure requiring penetrating keratoplasty, and then retinal redetachment requiring a second vitrectomy.

At 12 years of age, the patient had sudden vision loss in the left eye and was diagnosed with a second macula-involving retinal detachment (Fig. 3C “OS”). He underwent pars plana vitrectomy with endolaser and 5000 silicone oil tamponade. At last follow-up the patient's vision was no light perception in the right eye and counting fingers in the left.

The presence of bilateral retinal detachments and abnormal vitreous prompted genetic analysis. A commercially available microphthalmia, anophthalmia, coloboma, and anterior segment dysgenesis panel, as well as an inherited retinal disorders panel (Invitae Corporation, San Francisco, CA, CLIA-certified), were performed. The panel utilizes next generation sequencing, includes 330 genes for inherited retinal disorders and 81 genes for anterior segment disorders, and confirms variants not meeting stringent NGS quality metrics with Sanger sequencing. The patient was found to have two heterozygous pathogenic variants: *FOXC1* (c.-20_1125del, partial deletion in Exon 1) and *COL2A1* (Intron 27, c. 1833+1G > A, splice donor). This *COL2A1* variant has been previously described in dominant familial Stickler syndrome and leads to a premature termination codon and nonsense mediated decay of the mRNA.^12^^,^^13^ The patient was thus diagnosed with both ARS and Stickler Syndrome.

## 3 Discussion

The diagnosis of patients with multiple congenital pathologies presents a unique challenge, especially when the syndromes have overlapping clinical features. In the case of this patient, the clinical manifestations of ARS, as well as subsequent surgical management, complicated the diagnosis of Stickler Syndrome. Infantile-onset glaucoma results in buphthalmos and increased axial length, which increases the risk of retinal detachment. However, the clinician should be reminded that these are overlapping features with Stickler syndrome, which can manifest as infantile-onset glaucoma, while axial myopia may be confounded by buphthalmos. Given these overlapping features, it is possible that Stickler syndrome is underdiagnosed in childhood glaucoma patients, especially those with retinal complications. Thus, in cases where a glaucoma syndrome has been diagnosed (e.g., Axenfeld-Rieger syndrome), or when a glaucoma multi-gene panel revealed no pathogenic variants and the disease is attributed to primary congenital glaucoma or juvenile open angle glaucoma, additional testing for Stickler syndrome should be performed as the findings can inform future risks of retinal comorbidity and personalize surveillance.

Early in the patient's course, there was little suspicion for Stickler syndrome as vitreous stranding was not as apparent, there was no perivascular lattice, and the increased axial length was attributed to congenital glaucoma and his family history of high myopia. In retrospect, it could be argued that early genetic sequencing may have benefitted this patient, as prophylactic laser, scleral buckling, or cryotherapy could have been performed. However, despite some retrospective studies demonstrating favorable outcomes,^14^, ^15^, ^16^ laser prophylaxis for Stickler syndrome remains controversial. Reviews on this subject have argued that these studies are limited by their relatively small size, retrospective study design, and potential for bias.^17^ Thus, there is currently no consensus on the optimal type or timing of retinal detachment prophylaxis in Stickler syndrome. Further studies are needed in this regard.

Surgical repair of retinal detachment in Stickler syndrome is complex given the younger age of these patients, their propensity for developing multiple breaks or giant retinal tears, an abnormal vitreoretinal interface, and an increased incidence of proliferative vitreoretinopathy (up to 75% in some studies).^18^ There are multiple small case series describing surgical outcomes, but no randomized trial exists to date.^18^, ^19^, ^20^ One study found that patients required an average of 3.1 surgeries to achieve reattachment.^18^ For our patient, the presence of bilateral Baerveldt glaucoma tube shunts made the use of scleral buckles more challenging.

Management of this patient's glaucoma also presented a unique challenge given the degree and severity of pathology. Infantile glaucoma secondary to ARS often necessitates surgery.^1^ Goniotomy allows severing of irido-corneal adhesions under direct visualization and is often utilized as the first surgical intervention. However, in select cases, circumferential trabeculotomy may offer greater IOP lowering effect compared to goniotomy, though iatrogenic trauma in both approaches remains a concern given anatomical variations.^21^^,^^22^ A case series by Zepeda et al. examining surgical outcomes of glaucoma associated with ARS found that trabeculectomy with anti-fibrotics and Baerveldt glaucoma drainage devices were most successful in maintaining long-term IOP control.^1^ Other series advocate for primary combined trabeculotomy-trabeculectomy or inferiorly placed tube (as they require less frequent post-operative examinations under anesthesia and still allow for trabeculectomy at a later date if needed).^2^^,^^23^ Additional considerations in management of ARS include careful counseling regarding the risks of repeated anesthesia, adrenal crisis in patients with pituitary dysfunction, and intubation in patients with craniofacial abnormalities.^2^ Systemic manifestations of disease require referral to cardiology, endocrinology, craniofacial, and orthopedic specialists.

In conclusion, this is a rare case of concurrent Axenfeld-Rieger and Stickler syndromes in a single patient confirmed with molecular genetics. It highlights the unique challenge of diagnosing and managing multiple types of congenital pathology, and also demonstrates the advantages of genetic testing in the modern era. The surgical management of both diseases is highly complex, and in this case required close cooperation among the members of a multidisciplinary team. Proper counseling regarding the need for frequent exams under anesthesia, multiple surgeries, and poor visual prognosis are key, and systemic manifestations of the disease must be evaluated by appropriate specialists. Furthermore, proper genetic counseling regarding family planning can increase awareness of the possibility of producing an offspring with multiple rare genetic eye conditions.

## Funding/support

US National Institutes of Health Center Core Grant P30EY014801; an unrestricted grant to the University of Miami from the National Eye Institute; and Research to Prevent Blindness, New York, NY, USA (GR004596-1).

## Patient consent

Consent to publish the case report was obtained. This report does not contain any personal information that could lead to the identification of the patient.

## Funding

No funding or grant support.

## Authorship

All authors attest that they meet the current ICMJE criteria for Authorship.

## Declaration of competing interest

The authors declare that they have no known competing financial interests or personal relationships that could have appeared to influence the work reported in this paper.
